# Toxicity of enrofloxacin, copper and their interactions on soil microbial populations and ammonia-oxidizing archaea and bacteria

**DOI:** 10.1038/s41598-018-24016-8

**Published:** 2018-04-11

**Authors:** Ziyan Wei, Jinhua Wang, Lusheng Zhu, Jun Wang, Guodong Zhu

**Affiliations:** 1 National Engineering Laboratory for Efficient Utilization of Soil and Fertilizer Resources, Key Laboratory of Agricultural Environment in Universities of Shandong, College of Resources and Environment, Shandong Agricultural University, Taian, 271018 China; 2 Research Center for Eco-Environmental Sciences, Chinese Academy of Sciences, Beijing, 100085 China; 3 Jinan Science and Technology Information Institute, Jinan, 250001 China

## Abstract

Enrofloxacin (EFX) is one of the most frequently used broad-spectrum veterinary drugs, and copper (Cu) is a heavy metal that could easily bind to certain antibiotic molecules. Hence EFX and Cu were chosen as representatives of antibiotics and heavy metals to explore the abundance and variation of soil microbial populations with a plate-counting technique, as well as the copy numbers of *amoA* gene in ammonia-oxidizing archaea (AOA) and ammonia-oxidizing bacteria (AOB) by quantitative PCR methods in Argosols samples. Treatments of applied EFX (0.05, 0.20, 0.80 mmol/kg), Cu (1.60 mmol/kg) and combined EFX and Cu (at molar ratios of 1:32,1:8,1:2) exhibited various effects on different soil microorganisms. The toxicity of combined EFX and Cu was more strongly expressed on both soil microbial populations and *amoA* gene (AOA and AOB) compared to the EFX treatment alone, in most cases, time and dose effects were observed. With respect to the *amoA* gene, the AOA-*amoA* gene was more abundant than the AOB-*amoA* gene, and the ratio ranged from ~8 to ~11. Moreover, the interaction types of EFX and Cu were more likely to be antagonistic (64.29%) than synergistic (35.71%) on soil abundance and function, which may be related to the incubation time and the ratio of EFX to Cu in the soil.

## Introduction

Since their first discovery, antibiotics have played incomparable roles in treatment of diseases and promotion of animal feed efficiency^1^. There were more than 70 billion clinical doses of antibiotics employed globally in 2010^2^, and 162,000 tons of antibiotics were used in China in 2013, making up 48% and 52% of human and veterinary drugs, respectively^3^. Based on surveys, China has becoming the largest producer and user of antibiotics in the world^4,5^. The misuse or overuse of antibiotics results in their residues in various environments, such as soils^6^, agricultural farms^7^, wastewater treatment plants^8^, lakes^9^, rivers^10^ and groundwater^11^.

Heavy metals are essential for many organisms in trace amounts, but they may become toxic in high concentrations^12^. Coexistence of antibiotics and heavy metals, including copper (Cu), zinc (Zn), cadmium (Cd), chromium (Cr), and lead (Pb) have been detected with high concentrations in wastewater^13^, sewage treatment plants^14^, manures and soils^15^. Land application of wastes such as animal manure or reclaimed water as irrigation is a commonly adopted agricultural practice in many countries^16^, but this practice could accelerate the accumulation of antibiotics and heavy metal residues in farmlands. Hence farmland soil is one of the recipients and reservoirs that are most likely to be contaminated with both antibiotics and heavy metals. The combined accumulation of antibiotics and heavy metals in soils used for production of vegetables could be a threat to human health^17^. Antibiotics are deployed against a broad spectrum of bacteria, and high concentrations of heavy metals may also be toxic to microorganisms. It is thus hardly surprising that toxicity of antibiotics^18^ and heavy metals^19^ to microorganisms in soil has often been reported.

Aerobic ammonia oxidation plays a crucial role in the key nitrification processes of nitrogen (N) cycling. Ammonia-oxidizing organisms, including ammonia-oxidizing archaea (AOA) and ammonia-oxidizing bacteria (AOB), carry out this process by oxidizing ammonia-N to nitrite-N^20^. At first, AOB was thought to drive the ammonia oxidation process^21^, but recent studies have shown that AOA can also be detected with high abundance in soils^22,23^, suggesting that both AOA and AOB play pivotal roles in N cycling^23^. The functional *amoA* gene that encodes subunit A of ammonia monooxygenase exists in both AOA and AOB and is considered to be a fundamental biomarker for ammonia oxidation in soils^24^.

Enrofloxacin (EFX), one of the broad-spectrum synthetic quinolones, is most commonly used in China^3^, and its concentration could range from 0.3 to 1,421 mg/kg in manure^25^. Cu is one of the most common heavy metals present in metal-contaminated soils, and its concentration in soils has been regularly reported to reach 100 mg/kg in different countries^26,27^. Furthermore, Cu^2+^ may be bound to EFX through the carboxylate and pyridone moieties^28^. Therefore, in this study, we chose EFX and Cu as representatives of antibiotic and heavy metal contaminants to explore their effects alone, their combined effects and their interaction types on soil microorganisms in Argosols samples from Taian, China. Microbial abundance was measured in colony-forming units (CFU) of bacteria, fungi and actinomycetes. The abundance of the *amoA* gene in AOA and AOB was determined by quantitative PCR to indicate the response of an important microbial function. Here, our objectives are to answer three questions: (1) Do EFX and Cu, either alone or in combination, affect the abundance of living bacteria, fungi, and actinomycetes in consistent patterns? (2) Do EFX or Cu affect the abundance of ammonia-oxidizing genes in soil? (3) Does the molar ratio of EFX to Cu affect the degree of toxicity for different microbial communities in similar ways?

## Results

### General properties of the soil

The soil treated with different concentrations of EFX and Cu was from the surface horizon of a soil classified as Argosols. The properties of the Argosols samples were as following: clay content 10%, silt particle 57%, sand content 33%, pH 6.5, maximal field capacity 18.9 %, organic carbon 1.76 g/kg, total nitrogen 132.3 mg/kg, ammonium acetate extractable potassium 125.7 mg/kg, and sodium bicarbonate extractable phosphorus 16.5 mg/kg.

### Quantification of soil microbial populations

**Figure 1:**
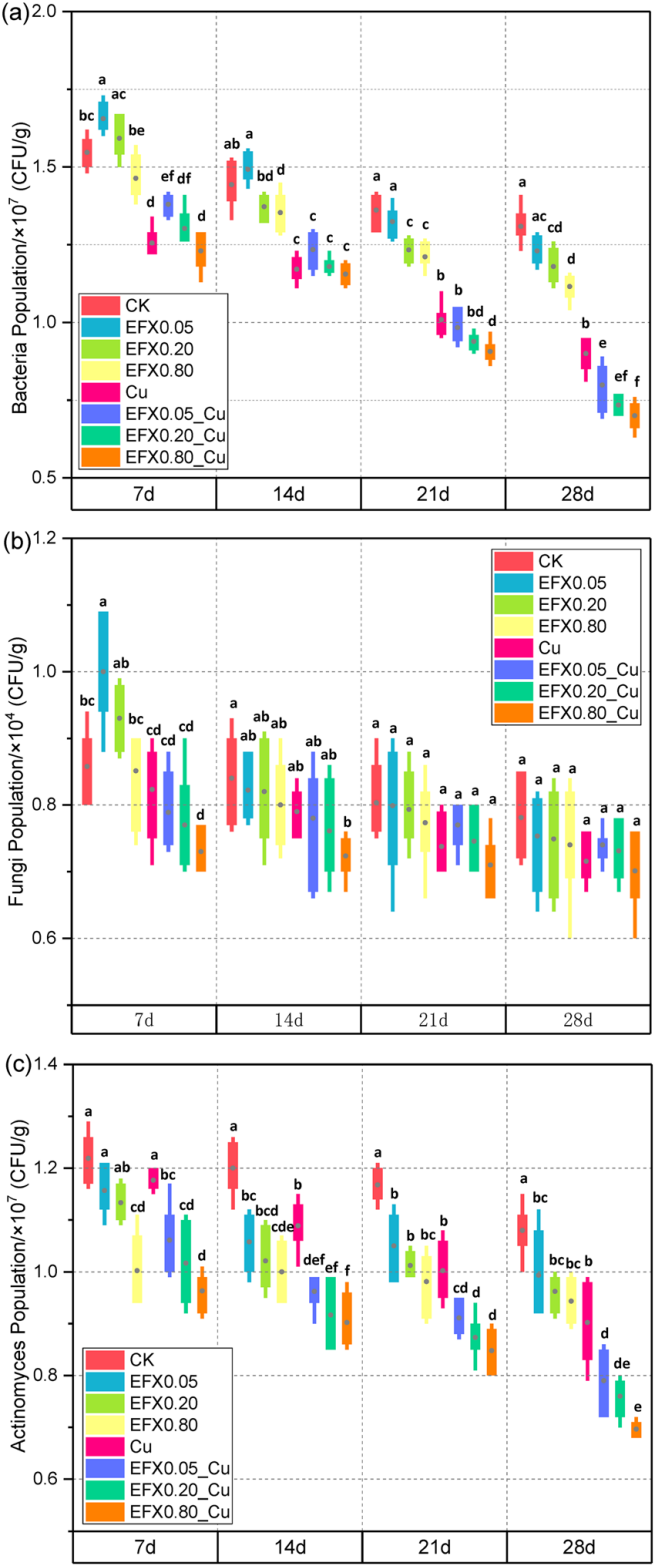
Variation in abundance of bacteria (**a**), fungi (**b**) and actinomycetes (**c**) under EFX and Cu treatments in 28 days.

The three microbial groups responded in different ways to the combined EFX and Cu, with an obvious reduction in the bacteria, fungi and actinomycetes numbers in most cases. The dynamic variation of soil microbial populations is displayed as box plots in Fig. 1. The toxicity of combined EFX and Cu on soil microbial populations was more strongly expressed compared to the EFX treatment alone, and the highest concentration of EFX and Cu treatment (EFX0.80_Cu) exhibited the greatest impact on soil microorganisms. Under this treatment, the bacterial populations in per gram soil were in the range of 7.00 × 10^6^–1.23 × 10^7^ CFU, fungal populations were 7.01 × 10^3^–7.30 × 10^3^ CFU, and actinomycetes populations were 6.97 × 10^6^–9.63 × 10^6^ CFU. While in the control soil, the number of microbial populations were ranked as follows: bacteria (1.31 × 10^7^–1.55 × 10^7^) > actinomycetes (1.08 × 10^7^–1.22 × 10^7^) > fungi (7.81 × 10^3^–8.58 × 10^3^) (CFU per gram soil). In brief, the cultural microbial populations all responded greatly after 28 days incubation with EFX and Cu, and the cultural fungal populations were three to four orders of magnitude less abundant than bacteria and archaea in the whole incubation.

The effects on soil bacteria of EFX alone and combined with Cu over the 28 days incubation period are displayed in Fig. 1a. Compared to the control soil, the bacteria reacted rapidly to EFX alone. The number of bacteria exhibited a significant (7-d) and an insignificant (14-d) increase at first, then an insignificant decrease happened after 21 days under the EFX0.05 treatment. The response of bacteria under higher concentrations of EFX (EFX0.20 and EFX0.80) showed insignificant differences at 7-d of incubation, and the inhibition effect became significant as the incubation time went on (*P* < 0.05). For the Cu (1.6 mmol/kg) treatment alone, there was a significant decrease (*P* < 0.05) in all the four periods. When they were combined, the count of bacteria showed a strong reduction with obvious time and dose effects.

Figure 1b showed the viability of fungal population in the presence of different EFX and Cu treatments for 28 days. The fungal population decreased, but not significantly, under the Cu treatment alone over the whole incubation period. The highest abundance of the fungal population occurred in the EFX0.05 treatment at 7-d of incubation, and the combined treatment of EFX0.08 and Cu significantly inhibited the growth of fungi. The number of fungi showed no significant differences among other single EFX or combined EFX and Cu treatments compared to the control after 14 days.

The quantification of actinomycetes under different treatments is shown in Fig. 1c. For the EFX alone treatment, the lower concentration (0.05 mmol/kg and 0.20 mmol/kg) showed insignificant inhibition on the growth of actinomycetes, while the high concentration treatment (0.80 mmol/kg) exhibited significant inhibition (*P* < 0.05) at the 7-d period. Then the actinomycetes population decreased significantly after 14 days of incubation. The number of actinomycetes under Cu treatment alone showed an insignificant decrease at 7 days and a significant decrease after 14 days compared to the control. The counts of actinomycetes were reduced with all combined EFX and Cu treatment, and there was dose effect on the variation of actinomycetes population.

### Abundance of AOA*-amoA* and AOB*-amoA* genes under different treatments of EFX and Cu for 21 days

**Figure 2:**
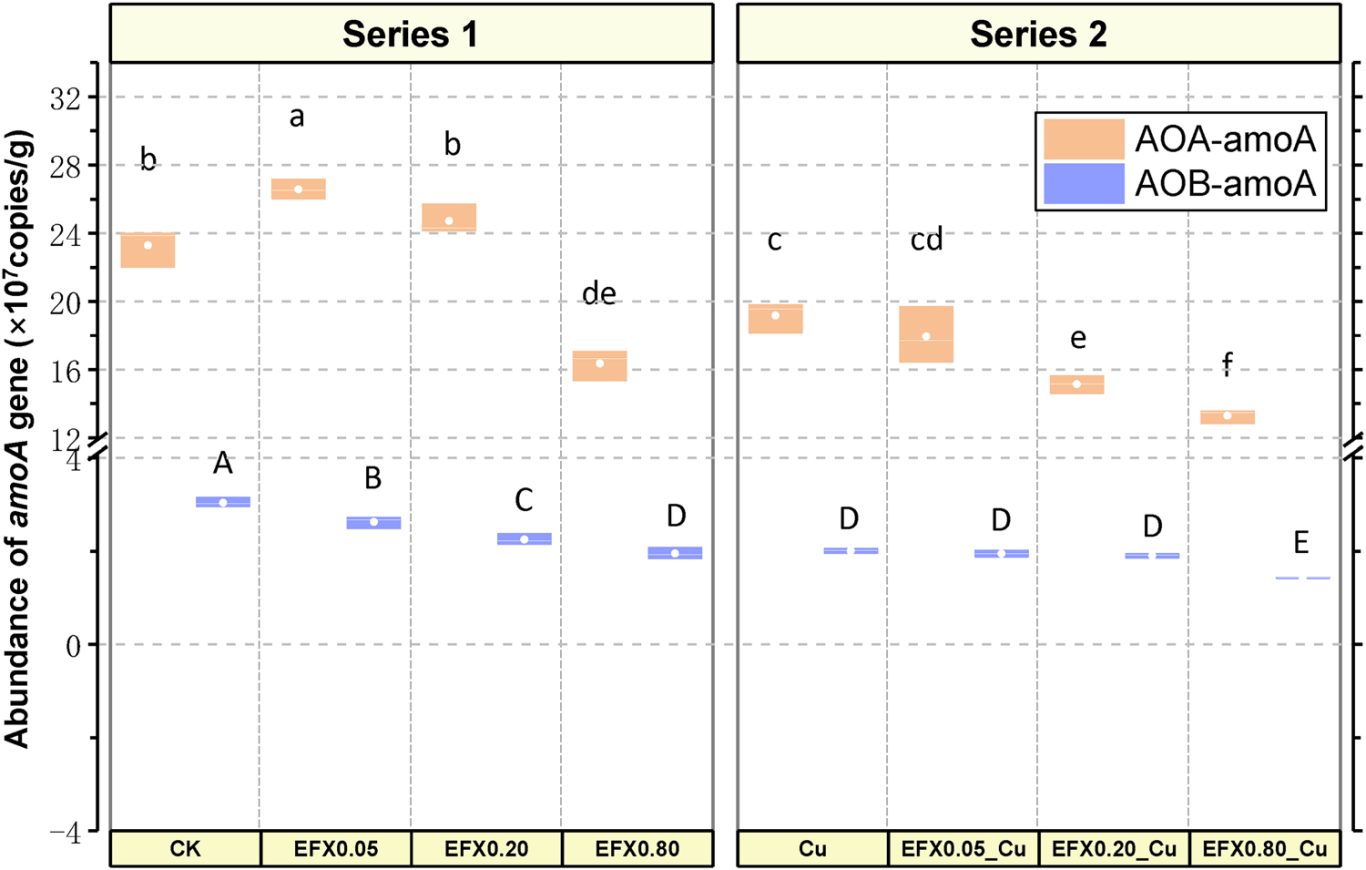
Box plot of abundance of AOA-*amoA* gene and AOB-*amoA* gene in soil samples treated with combined EFX and Cu for 21 days (Different letters a/b/c/d/e/f and A/B/C/D/E/F in columns are significantly different at *P* < 0.05 level between treatments on AOA-*amoA* gene and AOB-*amoA* gene respectively).

We then quantified AOA-*amoA* and AOB-*amoA* genes in soil after 21-d treatments of EFX and Cu. Indices of distribution and abundance of the *amoA* gene are displayed in Fig. 2. In total, the copy number of the AOA-*amoA* gene ranged from 1.33 × 10^8^ (EFX0.80_Cu) to 2.66 × 10^8^ (EFX0.05) copies per gram of soil, while the copies of AOB-*amoA* gene varied between 1.43 × 10^7^ (EFX0.80_Cu) and 3.04 × 10^7^ (CK) per gram of soil. Interestingly, the average copy numbers of AOA-*amoA* gene were one order of magnitude more abundant than those of the AOB-*amoA* gene, with the proportions of AOA to AOB genes ranging from ~8 (CK) to ~11 (EFX0.20), indicating the high abundance and wide distribution of the AOA-*amoA* gene in soil, even in the EFX and Cu treatments.

For the AOA-*amoA* gene (Fig. 2), the highest abundance occurred in the soil treatment with 0.05 mmol/kg EFX, indicating a promoting impact of EFX on the presence of the AOA-*amoA* gene at low concentration (EFX0.05). As the concentration of EFX rose, the abundance of AOA-*amoA* gene showed insignificant (EFX0.20, *P* > 0.05) and significant (EFX0.80, *P* < 0.05) decreases compared with the control. In Series 2, compared to Cu treatment alone, higher molar ratios (EFX0.20_Cu and EFX0.80_Cu) exhibited more significant inhibition on AOA-*amoA* gene. Such results indicated the dose-effect relationship of EFX and Cu on the abundance of AOA-*amoA* gene. In addition, the copy numbers of AOA-*amoA* gene in soil treated with combined EFX and Cu were significantly lower than those under the same concentration of EFX treatment alone, indicating that the effect on the AOA-*amoA* gene could be accelerated in the presence of Cu.

Soil treated with all concentrations of EFX alone and Cu alone showed significantly lower abundance of the AOB-*amoA* gene than the untreated soil (Fig. 2). In Series 2, the combined EFX and Cu treatments showed significantly higher inhibition than the EFX alone treatment at three levels, while compared to Cu alone treatment, only the highest molar ratio of EFX and Cu (1:2) exhibited significant differences. Such results suggested the extra Cu in Series 2 could combine with EFX and affect the abundance of AOB-*amoA* gene more. The overall trend in the abundance of AOB-*amoA* gene among the different treatments was that the copy numbers of AOB-*amoA* gene decreased as the concentration of EFX increased in both Series 1 and Series 2.

### Interaction type of EFX and Cu on the microbial population

According to the criteria proposed by Piggott, there are five potential types of interaction types of multiple stressors, including AD, +A, −A, +S and −S (refer to the Methods section for definitions of these acronyms)^29^. The interaction types of EFX and Cu can be identified from their relative influence, compared to their effects alone, on soil bacteria, fungi, actinomycetes, and AOA-*amoA*, AOB-*amoA*. The inhibition ratio was adopted as an index to reflect the single and combined toxicities of EFX and Cu on soil microorganisms (Tables 1 and 2).

**Table 1:** Interaction types of EFX and Cu on bacteria, fungi and actinomycetes indicated by inhibition ratio.

Microbe	Treatments	Inhibition Ratio (%)				Interaction types			
		7d	14d	21d	28d	7d	14d	21d	28d
Bacteria	Cu	18.82	18.86	25.88	31.24	—	—	—	—
	EFX0.05	−7.04	−3.39	2.69	6.03	—	—	—	—
	EFX0.20	−2.94	4.92	9.39	9.85	—	—	—	—
	EFX0.80	5.39	6.23	11.02	14.77	—	—	—	—
	EFX0.05_Cu	10.78	14.47	27.75	38.96	−A	−A	+A	+S
	EFX0.20_Cu	15.81	18.24	31.02	43.89	−A	+A	+A	+S
	EFX0.80_Cu	20.48	19.94	33.39	46.52	+A	+A	+A	+S
Fungi	Cu	4.02	5.95	8.16	8.39	—	—	—	—
	EFX0.05	−16.58	2.12	0.55	3.55	—	—	—	—
	EFX0.20	−8.42	2.38	1.24	4.12	—	—	—	—
	EFX0.80	0.78	4.76	3.73	5.26	—	—	—	—
	EFX0.05_Cu	8.03	7.14	4.15	5.26	+S	+A	+A	+A
	EFX0.20_Cu	10.24	9.39	7.19	6.40	+S	+S	+A	+A
	EFX0.80_Cu	14.90	13.89	11.61	10.24	+S	+S	+A	+A
Actinomycetes	Cu	3.46	9.26	14.18	16.46	—	—	—	—
	EFX0.05	5.10	11.85	10.09	8.02	—	—	—	—
	EFX0.20	7.02	14.91	13.32	10.91	—	—	—	—
	EFX0.80	17.78	16.67	15.98	12.65	—	—	—	—
	EFX0.05_Cu	12.94	19.81	21.98	26.85	+S	+A	+A	+S
	EFX0.20_Cu	16.59	23.61	25.21	29.63	+S	+A	+A	+S
	EFX0.80_Cu	20.97	24.81	27.40	35.49	+A	+A	+A	+S

**Table 2:** Interaction types of EFX and Cu on AOA-*amoA* and AOB-*amoA* genes indicated by inhibition ratio.

Treatments	Inhibition Ratio (%)		Interaction types	
	21-d (AOA)	21-d (AOB)	21-d (AOA)	21-d (AOB)
Cu	17.65	34.04	—	—
EFX0.05	−14.07	13.65	—	—
EFX0.20	−6.13	26.09	—	—
EFX0.80	29.74	35.91	—	—
EFX0.05_Cu	22.93	36.10	+S	+A
EFX0.20_Cu	34.95	37.47	+S	+A
EFX0.80_Cu	42.90	53.02	+A	+A

Judged on the basis of inhibition ratio, only antagonistic interactions (+A, −A) and synergistic interactions (+S, −S) were involved in the mixture of EFX and Cu. For soil bacteria, the interaction of EFX and Cu was negatively antagonistic at lower EFX:Cu molar ratios at 7-d (1:32 and 1:8) and 14-d (1:32) incubation, and became positively antagonistic at higher ratios or longer incubation times before 21 days, suggesting the interaction of EFX and Cu was stronger with higher concentration of EFX or longer incubation time, which was consistent with the toxic effect results. The toxicity of the mixture on actinomycetes functioned as a positively synergistic interaction at EFX:Cu ratios of 1: 32 and 1:8 at 7 days, and turned to positively antagonistic interactions with higher molar ratio (1:2) and longer incubation times (14-d and 21-d). After that, the combined toxicity acted as positively synergistic at 28-d, as it did for bacteria. On the other hand, the impact of combined EFX and Cu on fungi was predominantly a positively synergistic interaction at first, and then it changed to a positively antagonistic interaction after 14 days. Hence the interaction types on fungal population were very different from those on bacteria and actinomycetes in the soil samples.

For AOB-*amoA* gene, the interaction type of EFX and Cu was positively antagonistic at all molar ratios at 21 days. For the AOA-*amoA* gene, positively synergistic interaction occurred at the EFX:Cu molar ratios of 1:32 and 1:8, then shifted to positively antagonistic at the molar ratio of 1:2. There seemed to be no difference of interaction types between molar ratios of 1:32 and 1:8 within AOA-*amoA* or AOB-*amoA* gene. Such results suggested that the interaction types of EFX and Cu may be related to the proportions of EFX and Cu, the species of microbes as well as the incubation time.

## Discussion

Farmland soil is often the recipient of combined antibiotics and heavy metals, which could greatly influence soil microbial activity and function. We chose the frequently used and widely distributed EFX and Cu, two representative chemicals that could interact in soil, to explore the toxicity of single and combined EFX and Cu treatments on soil microorganisms. In this study, a biocenosis of soil microorganisms, including bacteria, fungi and actinomycetes, as well as AOA-*amoA* and AOB-*amoA* genes, was measured to assess the variations of microbial activity under different treatments of EFX and Cu. Moreover, we chose to use several molar ratios of EFX and Cu to better explore their interaction types in a soil.

The quantification results showed that microbial responses to EFX and Cu were highly heterogeneous due to the different capacities of the microbial communities for growth and adaptation. Single and combined EFX and Cu treatments exhibited different influences on microorganisms, and the influence was more significant for bacteria and actinomycetes as incubation time increased. In addition, the effect of EFX and Cu on microbes was more powerful with higher concentrations and higher EFX:Cu molar ratios. Overall, the effect of EFX and Cu was time dependent and exhibited a dose-response relationship. Effects of antibiotics on soil microorganisms have been previously shown to be correlated with incubation time and dosage^30^. In the present study, after 7 days of incubation, culturable bacterial and fungal populations under single EFX treatments ranked as follows: EFX0.05 > EFX0.20 > CK > EFX0.80, and the numbers of actinomycetes ranked as CK > EFX0.05 > EFX0.20 > EFX0.80. In addition, all microbial populations responded greatly at first, but at 21-d and 28-d incubation, significant reduction of bacteria and actinomycetes was still observed in most treatments, while the fungal numbers displayed no significant difference among all treatments. Previous studies also found the responses of bacterial and fungal activities varied at different antibiotic concentrations^30^.

In soil treated with EFX and Cu, the copy numbers of AOA-*amoA* and AOB-*amoA* genes were in the range of 1.33 × 10^8^ to 2.66 × 10^8^ and 1.43 × 10^7^ to 3.04 × 10^7^ copies per gram of soil, respectively. Similarly high abundance of the two genes has been reported in soils^20^ and other environments^31^. The abundance of the AOA-*amoA* gene and the AOB-*amoA* gene was reported in sediment with 7.49 × 10^6^–2.07 × 10^8^ and 3.96 × 10^6^–2.66 × 10^7^ copies per gram^31^, and in soil with 1.5 × 10^7^–1.7 × 10^7^ and 1.1 × 10^4^–1.0 × 10^7^ copies per gram^20^. Generally, the abundance of the *amoA* gene in the present study was comparable to previous studies, reflecting its frequent and prevalent occurrence in soil environments. The response of AOA-*amoA* and AOB-*amoA* genes to different treatments of EFX and Cu varied. Overall, a significant reduction occurred in copy numbers of AOA-*amoA* and AOB-*amoA* genes with combined EFX and Cu compared to single EFX treatments. In particular, the abundance of AOA-*amoA* gene copy numbers increased under EFX0.05 and EFX0.20 treatments, while the abundance of AOB-*amoA* gene copy numbers decreased significantly compared to the control treatment. Previous studies have also observed variations in the abundance of AOA-*amoA* and AOB-*amoA* genes with diverse conditions^31,32^.

Although AOA-*amoA* and AOB-*amoA* genes were both abundant, AOA showed significant higher copy numbers than AOB. The ratios of AOA to AOB copy numbers ranged from ~8 to ~11, indicating the dominant role of AOA in the studied farmland soil under EFX and Cu treatments. This result is consistent with the reported dominance of AOA in soil and other environments^33,34^. However, the roles that AOA or AOB play in nitrogen cycling in different environments is still debated. In some reported studies, AOB predominated over AOA numerically, and AOB were thought to be more important in the nitrification process^31,32^.

The combination of EFX and Cu inhibited microbial activity more than single EFX and Cu treatments in most cases. But the interaction types varied among the different kinds of microorganisms or at different concentrations and different incubation periods. For fungi, a significant difference was found only between the EFX alone and the combined EFX and Cu treatments at 7-d of incubation, and their interaction type was synergistic at first; then it shifted to antagonistic. The combined effect of EFX and Cu on bacteria turned from antagonistic to synergistic interaction, which was very different from that of fungi. The interaction on actinomycetes was similar to that on fungi at first, i.e., synergistic then antagonistic, but it shifted to synergistic at last. Such results suggested that fungi may be not as sensitive to EFX or to Cu at these concentrations as bacteria or actinomycetes. In total, the combined effect of EFX and Cu on the tested soil microorganisms acted as antagonistic (64.29%) more frequently than synergistic (35.71%) in laboratory conditions. The variable interaction types indicated that the interaction of EFX and Cu may be related to the ratio of EFX to Cu, incubation period^35^, and other stressors^29,36^.

The soil properties may also play important roles in shaping the interactions of EFX and Cu^37^. Soil pH is one of the most important factors affecting the interaction of EFX and Cu, since EFX was present in different forms with different pH, such as zwitterionic form at pH 6 - 7.7, and anionic form above pH 7.7^37^. The changeable forms could then affect the binding affinity of EFX and Cu^37^. Previous studies found that compared to Cd and Pb, Cu could more easily bind to tetracycline especially when pH < 7, and the release of tetracycline from the soil could induce the release of Cu from soil at pH > 7^38^. In addition, the combined effect of Cu and oxytetracycline/captan on soil respiration rates was found to be highly affected by the concentration of Cu and soil properties^39^. According to Wyszkowska^40^, combined heavy metals (Ni and Zn, Cu, Pb, Cd, Cr) may either inhibit or stimulate the growth of soil microbes, depending on the type of microbes, the ratios and dose of heavy metals, as well as the soil texture, such as loamy sand or silt loam. In addition, soil bacterial communities have shown variable responses with treatments of Cd, Zn and phenanthrene over different incubation periods^41^. Furthermore, other soil properties like organic matter, iron and manganese oxides, and temperature may also influence the impacts of metals on soil microorganisms^42,43^.

The ultimate aim of our studies is to integrate the known interaction types of antibiotics and heavy metals at different proportions into real-world models to better manage their effects on microbial activities and functions. However, there are always many stressors in natural ecosystems, making it difficult to measure their interactions with certainty in different environments^44,45^. Therefore, in future studies it is necessary to take into account environmental variables. The mechanisms of interaction among antibiotics, heavy metals and other stressors in the same environment should also be further studied to provide key guidance that is consistent with theoretical interactions.

Overall, we demonstrated that EFX and Cu have a strong and variable influence on soil microbial populations. Under the conditions of this experiment, the toxicity of EFX and Cu was similar and significant for bacteria and actinomycetes compared with fungi. The AOA-*amoA* gene was more abundant than the AOB-*amoA* gene under EFX and Cu treatments after incubation for 21 days. The interaction types of combined EFX and Cu were dominated by antagonistic rather than synergistic interaction in this designed experiment. But they were also variable in different conditions, indicating the difficulty in understanding the interactions between antibiotics and heavy metals in natural environment with more uncertainties. Overall, these data can be used in phylogenetic or multiple stressor frameworks for prediction and generalization of responses to antibiotics and heavy metals in the future.

## Methods

### Chemicals and source of soil

Enrofloxacin lactate (EFX) (99.0 %, purity) was purchased from HETIAN Biotech Co., LTD (Zhengzhou). Copper sulfate (Cu) (99.0 %, purity) was purchased from YONGDA Chemical Reagent Co., LTD (Tianjin). LB broth agar was purchased from Sangon Biotech (Shanghai) Co., Ltd. Other chemicals used for the culture medium, including glucose, sodium chloride, magnesium sulfate, ferrous sulfate, dipotassium phosphate, and sodium nitrate were of analytical grade and purchased from Sinopharm Chemical Reagent Co., Ltd.

The experiments were performed with topsoil (0–20 cm) from a peanut experimental field. The soil samples classified as Argosols were collected from Shandong Agricultural University in Taian, China (117.16°E, 36.16°N) during the slack farming seasons in November, 2012. This soil had never been treated with organic fertilizer or exposed to heavy metals. The soil samples were air-dried and ground to pass a 2-mm sieve to remove stones and plant roots. Soil physical and chemical parameters were measured according to the soil agro-chemistrical analysis methods^46^.

### Experimental design

The soil was pre-incubated at 25 °C for 7 days to allow biological activity to recover, and soil moisture was maintained at 60% of the maximal field capacity (11.34%) in the whole incubation. In the Series 1 experiment, soil samples were exposed to 0.1 mL of EFX solution with several concentrations of 4.43, 17.73, 70.93 mmol·L^−1^ to generate the final molar concentration of EFX at 0.05, 0.20, 0.80 mmol·kg^−1^ dry soil and no Cu^2+^ were added. In Series 2, soil samples were treated with the four levels of EFX solution as Series 1, additional 0.1 mL of Cu^2+^ at 141.86 mmol·L^−1^ were added to make the molar concentration at 1.60 mmol·kg^−1^ dry soil, hence the molar ratio of EFX to Cu^2+^ was 1:32, 1:8, 1:2 (Table 3). Each treatment consisted of 13 replicates (3 replicates at each period for the colony-forming units (CFU) calculation, and one at 21-d for DNA extraction), and 10.00 g soil were treated in each replicate following Table 3. The treated soil samples were incubated in sealed amber bottles at 25 °C for 4 weeks in dark under micro-anaerobic condition. Soil moisture content levels were maintained by weighing and adjusted to 11.34% by adding sterile water every 2 days. Soil samples were collected in triplicate at 7, 14, 21 and 28 days (every 7 days) for the CFU calculation of soil bacteria, fungi and actinomycetes. After incubated for 21 days, soil samples were also collected for the quantification analysis of AOA-*amoA* gene and AOB-*amoA* gene.

**Table 3:** Treatments of different concentrations of EFX and Cu (mmol per kg dry soil) in soils.

Series	Treatments	EFX	Concentration (mmol/kg)	Cu^2+^	Concentration (mmol/kg)	Mol Ratio (EFX: Cu^2+^)
Series 1	CK	−	0	−	0	−
	EFX0.05	+	0.05	−	0	−
	EFX0.20	+	0.20	−	0	−
	EFX0.80	+	0.80	−	0	−
Series 2	Cu	−	0	+	1.6	−
	EFX0.05_Cu	+	0.05	+	1.6	1:32
	EFX0.20_Cu	+	0.20	+	1.6	1:8
	EFX0.80_Cu	+	0.80	+	1.6	1:2

### Culture Medium and culture conditions

Soil samples were collected at 7-d, 14-d, 21-d, and 28-d incubation and immediately analyzed. The entire 10.00 g sample of soil was added to 90.00 mL of sterile water, homogenized by shaking at a rate of 200 rpm for 20 min, let sit for 5 min. The concentration of the soil supernatant was 10^−1^ g/mL (1:10 dilution), and serially diluted to 10^−4^ concentration. Bacteria, fungi, and actinomycetes were cultured using Luria-Bertani (LB)^47^, potato dextrose agar (PDA) with bacteria inhibitor - penicillin and streptomycin^48^, and Gauserime synthetic agar medium with potassium dichromate to suppress other bacteria^49^, respectively, with 100 μL diluted soil suspension (10^−4^) by rubbing method. Each type of medium was treated in triplicate with the same suspension, hence there were nine replicates for each treatment at each period. Incubation of bacteria, fungi, and actinomycetes was carried out in the media at 25 °C for 36 hours, 48 hours, and 5 days, respectively, in a biochemical incubator. The numbers of colonies were counted at the end of the incubation period. The average counts of the nine plates and the corresponding dilution ratio were used to calculate the abundance of soil bacteria, fungi, or actinomycetes.

### DNA Extraction and quantification of *amoA* gene by quantitative PCR

From 0.50 g homogenized soil of each 21-d cultured treatment, we extracted DNA with the PowerSoil^®^ DNA isolation Kit (MO BIO Laboratories, San Diego CA), according to the manufacturer’s directions. Genomic DNA was detected by agarose gel electrophoresis and quantified with NanoDrop 2000 (Nanodrop, USA).

Quantitative PCR (qPCR) was performed to determine the absolute abundance of *amoA* gene in AOA and AOB from total DNA samples using CFX96 Touch Real-Time PCR Detection System (BioRad). Primer sets Arch-amoAF and Arch-amoAR^50^, amoA-1F and amoA-2R^51^ were chosen for the quantification of *amoA* gene in AOA and AOB, respectively. Standard curves were generated by a ten-fold dilution series of the standard plasmids carrying the targeted amplicons, with R^2^ value higher than 0.99, and proper amplification efficiencies (90–110%). Each 20 μL reaction mixtures typically contained 1× SuperReal PreMix Plus (TianGen, China), 0.3 μM of each primer, and 20 ng of DNA. Each sample contained technical triplicates with parallel negative controls. The qPCR program consisted of initial denaturing for 15 min at 95 °C, followed by 40 cycles of 10 s at 95 °C, 30 s at annealing temperatures (55 °C for AOA-*amoA* gene or 60 °C for AOB*-amoA* gene), and a final melt curve stage with temperature ramping from 55 °C to 95 °C (0.5 per read, 5 s hold).

### Ecological and Statistical Analysis

Based on the abundance of soil bacteria, fungi, actinomycetes, and quantification of AOA*-amoA* and AOB-*amoA* genes, ANOVA was performed in SPSS to determine the significance of differences among treatments of EFX and Cu at different incubation times. Box plots (the range of box and whisker was 20–80%, 0–100%, respectively; and the dots represented mean values) were adopted to display the variations of microbial abundance, as well as the different distribution of AOA-*amoA* and AOB-*amoA* genes under EFX and Cu treatments.

When two stressors are combined, taking into account of the magnitude and response direction (positive (+) or negative (−)), there could be five interaction types: additive effect (AD: the sum of individual effects), positive antagonistic interaction (+A: less positive than predicted antagonistic, the interaction is lower than AD or no more than any individual effect in the same direction), negative antagonistic interaction (−A: less negative than predicted antagonistic), positive synergistic interaction (+S: more positive than predicted synergistic, the interaction is higher than AD, greater than any individual effect in the same direction or than the absolute individual effect in both directions), and negative synergistic interaction (−S: more negative than predicted synergistic) (Table 4)^29^. The Inhibition Ratio (IR) was used as an index of the differential toxicity and interaction types of EFX and Cu (Eq. (1)).

1 $${\rm{IR}}=\frac{{\rm{Unamended}}\,{\rm{Population}}-{\rm{Treated}}\,{\rm{Population}}}{{\rm{Unamended}}\,{\rm{Population}}}\times 100 \% $$

**Table 4:** Potential interaction types of two chemicals. The individual chemical effect is classified directionally: positive direction (+) and negative direction (−).

Interaction Type	Individual effect	Combined effect	Definition
AD	++	+	Interaction = AD
	−−	−	Interaction = AD
	+−	0	Interaction = AD
+A	++	+	0 < Interaction < AD
	++	−	Interaction < 0 < AD
	+−	−	Interaction < AD < 0
−A	−−	−	Interaction < AD < 0
	+−	+	0 < Interaction < AD
+S	++	+	0 < AD < Interaction
	−−	+	AD < 0 < Interaction
	+−	+	AD < 0 < Interaction; 0 < AD < Interaction
−S	−−	−	AD < Interaction < 0
	+−	−	AD < Interaction < 0
